# Physical and social availability of alcohol for young enlisted naval personnel in and around home port

**DOI:** 10.1186/1747-597X-2-17

**Published:** 2007-06-30

**Authors:** Roland S Moore, Genevieve M Ames, Carol B Cunradi

**Affiliations:** 1 Prevention Research Center, Pacific Institute for Research and Evaluation, 1995 University Avenue, Suite 450, Berkeley, CA 94704, USA; 2 School of Public Health, University of California, Berkeley, California, USA

## Abstract

**Background:**

Heavy alcohol consumption rates are higher in the young adult military enlisted population than among civilians of the same age. The literature on alcohol availability, both generally and specifically with respect to work-related drinking, establishes clear links between ease of access, alcohol consumption rates and alcohol-related problems.

**Methods:**

In this paper, a qualitative analysis of 50 semi-structured interviews with U.S. Navy personnel was combined with quantitative findings from a mail survey of 713 young enlisted men and women in order to shed light on alcohol availability and its correlates in the home port environments of young adult enlisted personnel. The interviews were analyzed by two anthropologists seeking recurring themes or topics related to alcohol availability. These qualitative data were contextualized by descriptive statistics of the survey responses regarding ease of obtaining alcohol on and around naval bases, and from friends in and out of the Navy.

**Results:**

Findings associated with social and physical availability of alcohol include low prices in Navy Exchange base stores, frequent barracks parties, drink promotions in bars surrounding bases, and multiple opportunities for underage drinking despite age limits on alcohol purchases and official efforts to deglamorize alcohol use in the Navy. Both qualitative and qualitative findings suggest that respondents found alcohol and opportunities to drink overwhelmingly available in both on-base and off-base settings, and from friends both in and out of the Navy.

**Conclusion:**

There is qualitative and quantitative evidence for extensive physical and social availability of alcohol in and around bases for young adults in the military. Policy implications include raising the presently tax-free alcohol prices in base stores and enforcing existing policies regarding underage drinking, particularly the provision of alcohol by people of legal drinking age, and by bars in and around bases. Cooperative preventive efforts with surrounding communities also offer promising ways for bases to reduce alcohol availability for young adult servicemembers.

## Background

As the United States Department of Defense (DoD) Surveys of Health Related Behaviors among Active Duty Military Personnel [1] indicate, heavy alcohol consumption rates are higher in the young adult enlisted population than among civilians of the same age. It is worth noting that the military draws many of its applicants from low socioeconomic status populations, with comparatively high-risk drinking patterns [2], and many of the underage recruits who already have extensive drinking histories bring their established drinking patterns with them to boot camp [3]. Accordingly, the extent to which alcohol-related problems are found in the military are disproportionately high [4]. The historical roots of alcohol's symbolic and instrumental roles in the military are deep-seated and include the grog or rum rations allotted to sailors in the British and U.S. Navies. Traditions and rituals around alcohol consumption and life at sea have gone hand in hand for centuries, as has easy availability of alcohol starting with the daily grog ration on board ships in the 18th and 19th centuries [5] right up to contemporary times where the multiple bars in port and on deployment liberty support normative patterns of heavy and binge drinking [6].

The U.S. Navy's tolerance of problematic drinking behavior began to decrease 35 years ago in response to heavy alcohol and drug use among troops during the Vietnam War [7]. From 1972 onward, the Department of Defense has issued a series of policy directives aimed at prevention and treatment of all substance abuse among military personnel in the Army, Navy, Marines, and Air Force [8]. The zero tolerance policy for drugs was indeed effective in lowering rates of illicit drug use from 36.7% in 1980 to 6.9% in 2002, but heavy alcohol use has remained fairly constant between 1980 and 2002: 20.8% in 1980, 24.8% in 1985, 20% in 1988, and essentially unchanged through 2005 [1]. These statistics indicate that the military's policies and education programs regarding alcohol problems, while positive in intent, have not been all that successful in curbing heavy and binge drinking.

Despite the fact that alcohol rations were abolished in the U.S. Navy in 1862 [5], drinking continues to play an integral role in the lives of enlisted personnel in the Navy. A key facilitator of drinking in military occupational culture, both in the past and the present, has been the ready availability of alcohol to members of the military.

In the alcohol research literature, availability theory contends that elevated levels of physical availability and social availability of alcohol generally increase both rates of consumption and consequent alcohol problems. The literature on alcohol availability establishes clear links between ease of access (availability), alcohol consumption rates and alcohol-related problems [9-15]. To test the theory, natural experiments in which the availability of alcohol has been reduced (e.g., supplier strikes) have demonstrated attendant reductions in alcohol consumption and alcohol-related harm [16].

*Physical availability *refers not only to the extent to which alcohol is accessible in a given environment but also to the barriers or costs associated with obtaining it [17]. National, state, and local laws and other policies (including military regulations) offer a variety of restrictions on who may purchase or consume alcohol, how much they pay for it, and when and where they may buy or drink it. A distinction can be made between objective and subjective physical availability of alcohol [15]. *Objective *physical availability refers to the actual legal, organizational, and geographical factors that affect the costs in time and/or money to acquire alcohol. Studies on the relationship between objective physical availability and general population drinking have focused on issues such as outlet density [12], and drinking age [18]. Price is another component of availability that modifies consumption and consequent harms of alcohol [19,20]. *Subjective *physical availability refers to perceptions of these factors, including beliefs about ease or difficulty of obtaining alcohol. Previous research [9,10] indicates that such beliefs influence drinking patterns.

*Social availability *refers to the degree of normative support for or against drinking within one's social environments. Whereas objective social availability refers to the actual drinking of family, friends and social referents, subjective social availability consists of an individual's perceptions of drinking norms in a given environment. Subjective social availability is thus similar to theoretical constructs such as the perceived environment [21], normative beliefs [22], and peer behavior [23].

Alcohol availability has been found to be equally important in occupational settings [24]. Refining the descriptions of physical and social availability for the workplace setting, Ames and Grube defined "*physical availability of alcohol at work *as the ease or difficulty with which an individual can obtain alcohol for work related consumption," and "*social availability of alcohol in the workplace *as normative support for work-related drinking within one's work environment" ([24]:384). More specifically, the physical availability of alcohol at work includes procuring alcohol in or around work sites or events, the rules or policies restricting its purchase, possession, and use, and the extent to which these rules or policies can be enforced. The social availability of alcohol at work may be indexed by the frequency of work-related drinking by one's friends or immediate co-workers, and the approval or disapproval by one's work friends, co-workers and supervisor of a worker's drinking on the job.

Work-related *social *availability of alcohol is subject to prevailing norms (in and around the workplace), whereas work-related *physical *availability of alcohol is constrained largely by social controls that take the form of policy, regulations, informal guidelines and enforcement of these rules, as well as geographical factors such as the clustering of alcohol outlets around the workplace. Work-related social and physical availability overlap in that the prevailing norms around alcohol help policymakers shape the controls over alcohol and demand for and distribution of alcohol, which in turn affect norms. Moreover, mandated alcohol and drug testing for certain occupations such as transit workers [25] and labor relations issues touching on alcohol use before or during work hours [26] may influence norms supporting or inhibiting work-related drinking.

Alcohol consumption is not permitted aboard U.S. Navy ships, with the rare exceptions of ceremonial drinks (e.g., to honor a visiting high-ranking officer or a Captain's retirement while the ship is docked) and tightly controlled beer distribution (2 per crewmember) during "steel beach picnics" when vessels have been at sea for at least 45 consecutive days. Thus, the availability of alcohol discussed in this paper refers to the drinking opportunities in the ports to which enlisted personnel are assigned, rather than aboard ships, submarines, or naval aircraft. Leisure time apart from working hours and time on duty (standing watch) is termed liberty for enlisted personnel, and it is within the framework of liberty time that enlisted crewmembers engage in recreation, which in the absence of other compelling activities may often include alcohol by default.

It is important to note that the evidence suggests that the vast majority of drinking by members of the military occurs during off-duty time. However, as a perpetually on-call occupation, the division between work and leisure for the military is not as clear-cut as it is for other professions, and it can be argued that all drinking by military personnel, whether on or off duty, is in fact work-related due to the fact that they are subject to be called to duty at any time. Another factor affecting the overall availability of alcohol in the military is the mandatory random drug testing that has been administered to military personnel since 1981 [27]. To the extent that the use of intoxicants other than alcohol may result in expulsion from the military, alcohol becomes the most attractive intoxicant of choice.

Based on our previous work on alcohol availability with factory workers [24], we hypothesized at the outset that high physical and social availability of alcohol in the occupational environment of young adult military members would contribute to higher rates of heavy alcohol consumption. We have reported elsewhere on availability issues within the deployment liberty environment [6]. The goal of this paper is to shed light on the dimensions of work-related alcohol availability and its correlates in the home base environments of young adult enlisted men and women in the U.S. Navy.

To contextualize the settings and population discussed below, it is worth briefly describing the numbers of bases and personnel affected by alcohol availability issues in and around home ports. Although there are over 50 Navy commands located throughout the U.S., and bases in at least 17 countries, there are 14 major areas within the US (including Bangor, Bremerton and Everett, WA, Groton, CT, Ingleside, TX, Little Creek, VA, Mayport, FL, Newport News and Norfolk, VA, Pascagoula, MS, Pearl Harbor, HI, Portsmouth, NH, and San Diego, CA) and 6 overseas (including Manama, Bahrain, Gaeta and La Maddalena, Italy, Guam, and Sasebo and Yokosuka, Japan) in which U.S. naval vessels are homeported [28]. San Diego and Norfolk are by far the largest of these homeports. As of April 9, 2007, the number of active duty personnel in the U.S. Navy numbered 341,385, including 51,169 officers, 4364 midshipmen, and 285,852 enlisted personnel. Over 60,000 of these naval personnel were on deployment [29].

## Methods

The methods by which we assessed work-related alcohol availability include the use of the ethnographic textual management system ATLAS.ti [30] to identify recurring themes concerning alcohol availability on and around bases from semi-structured interviews with 50 U.S. Navy personnel, and basic statistical comparisons of responses to questions about ease of access to alcohol from a mail survey of 713 young adult enlisted U.S. Navy personnel. Although definitions of young adulthood vary in their upper limit, we used the age range of 18 to 29 as the focus of our study; almost all enlisted study participants fell within this range.

### Ethnographic methods

In the context of a larger study on military drinking, we collected 50 semi-structured, open-ended interviews with a quota sample of 24 enlisted young men and women, 7 junior officers and 19 key experts including medical personnel, alcohol counselors, master chiefs and line officers. These hour-long face-to-face interviews were carried out on five large military bases in the United States: two in the East Coast (Norfolk, VA and Jacksonville, FL), one on the Gulf Coast (Pensacola, FL), one on the West Coast (San Diego, CA), and one in the South Pacific (Pearl Harbor, HI); Another dozen interviews were conducted at a U.S. base in Southern Europe (Naples, Italy). The drinking age in and around each of these bases was 21 except in Naples, where the DoD-specified limit of 18 applied on base and the local age limit of 16 applied off-base. At each of these bases, we asked medical officers to help us identify knowledgeable key informants as well as pools from which we randomly selected young adult respondents representing a variety of occupational specialties. We then asked the individuals we selected if they were willing to take part in an interview. The voluntary and confidential interviews were conducted out of earshot of other naval personnel in shore offices as well as in private work and recreational areas on board ships.

The semi-structured instruments guiding the interviews included questions on alcohol use by peers, prevailing norms surrounding alcohol use on and off base, respondent understandings of alcohol control policy and enforcement on and off base, and aspects of occupational culture that the respondents could relate to patterns of alcohol consumption. The interviewers would also ask clarifying questions or probes, in order to better understand the respondent perspectives on the issues discussed.

Additionally, informal observations of drinking establishments and off-premise outlets in and around the bases were conducted in order to gain a better understanding of the physical environments for obtaining alcohol.

### Quantitative methods

The quantitative data reported on here are derived from a sample consisting of 713 enlisted Navy personnel who completed a second wave of survey follow-up for a longitudinal study on changes in drinking patterns and problems among young adults in the military. They had originally completed a group-administered paper and pencil survey two years prior at the Recruit Training Center in Great Lakes, IL with a response rate of 93% [3]. Those who had enrolled at baseline in 1998 and were still on active military duty in 2000 were mailed a self-administered questionnaire. A cover letter was enclosed reminding them of their initial participation in the study, and asking them to once again participate by filling out the follow-up survey. The purpose of the study, and the voluntary nature of their participation, was repeated. A postage-paid business reply envelope and a pre-paid thirty-minute phone card incentive to complete the follow-up survey were included in the mailing. A reminder postcard was mailed to each participant one week following the first mail out. Up to three attempts per participant were made to elicit a completed survey. The follow-up response rate was approximately 40%; this response rate, although low, is similar to those seen in other mailed military survey studies [1,31]. (As a specific point of comparison, the mailed portion of the most recent World Wide survey to remote Naval personnel had a 33.3% response rate, in contrast to higher group administration response rates [1]) The surveys were confidential rather than anonymous, because we were attempting to link individual self-reports at the two waves of the survey.

Of the 713 follow-up respondents, 493 were male and 220 were female. There were significant gender differences in the race/ethnicity of the respondents; most men were white (56.6% White; 17.0% Black; 14.1% Hispanic; and 12.3% Other), and most women were non-white (42.6% White; 25.9.0% Black; 15.7% Hispanic; and 15.7% Other). Approximately one-quarter of the men, and one-third of the women, were married or cohabiting (26.8 and 36.6%, respectively). The mean number of years of education was 12.2 (0.55) for the men and 12.4 (.90) for the women. The mean age at follow-up among the enlisted respondents was 21.3 (1.9) years for the men and 21.5 (2.4) years for the women; most were underage at the time of the follow-up survey.

Survey participants were asked questions about their usual quantity and frequency of beer, wine, liquor, and other alcoholic beverage consumption in the previous 12 months. In accord with previous research on gender-based heavy episodic drinking measures [32], we classified males who reported drinking at least five drinks per typical drinking occasion at least once a week, and females who reported drinking at least four drinks per typical drinking occasion at least once a week, as frequent heavy drinkers. Using these measures, 26.8% of the men and 12.5% of the women in this sample were classified as frequent heavy drinkers [33].

We conducted a non-response analysis, which is discussed in detail elsewhere [34]. However, multivariate logistic regression analysis indicated that men, participants who were age 29 or less at baseline, and tobacco users at baseline were significantly more likely than females, those aged 30 or older at baseline, and non-tobacco users to be non-responders at follow-up. In addition, a significant interaction between level of education and baseline drug use indicated that respondents with less than a college education who were drug users were more likely to be non-responders.

### Survey measures

The survey items concerning subjective availability of alcohol asked respondents to rate on a four-point scale (from "very easy" to "very difficult") how easy or difficult they thought it was to obtain alcohol in a variety of situations, including on base after work, off base after work, from friends in the Navy, and from friends outside of the Navy.

### Human subjects approval

The overall research design, consent forms and other research materials were approved by the Institutional Review Boards of the US Naval Health Research Center and the Pacific Institute for Research and Evaluation.

### Analysis

The ethnographic interviews were tape recorded, transcribed, and entered into the ATLAS.ti qualitative analytical software program [30]. Following the thematic coding principles laid out by Ryan and Bernard [35], we coded the interviews for recurring themes or topics. In our thematic analysis, examples of important factors for physical and social availability of alcohol included the following themes: (1) Low drink prices, especially in on-base exchanges, (2) opportunities to drink at bars around bases, and (3) multiple opportunities for underage enlisted naval personnel to obtain alcohol. Using ATLAS.ti, we retrieved the coded segments of text concerning availability and examined other codes that were assigned to the same and adjacent segments of text. A small subset of respondent quotes illustrates recurring themes in this article. Selection criteria for these quotes included clear articulation of these themes, eloquence and brevity. Queries to the enlisted personnel are represented within brackets, and are included when necessary to frame their answers.

Descriptive statistics were used to summarize the relevant survey results. Chi-square tests were used to determine if there were statistical differences between the respondents who were 20 and younger vs. those who were 21 and older at the time of the follow-up survey. All quantitative analyses were conducted using SPSS v.11.0.1 [36].

## Results

The analysis of ethnographic interviews with young adults in the U.S. Navy revealed recurring themes associated with physical and social availability of alcohol, including major domains which will be covered in the following four sections:

1) Social availability in and around Navy bases;

2) On-base aspects of physical availability including lower prices in Navy Exchange base stores, and on-base social availability of alcohol as a default recreational activity;

3) Off-base opportunities for drinking, including the clustering of bars surrounding some bases; and

4) Underage servicemembers' special circumstances pertaining to the physical and social availability of alcohol.

The interview excerpts that are presented below serve as examples of frequently stated perceptions by naval enlisted personnel concerning aspects of access to alcohol, both on and off the military bases on which they work.

### 1) Social availability in and around bases

Although there are many barriers to underage drinking on the Navy bases we studied, most respondents revealed that the social availability of alcohol among young adults in the military occupational culture was high – whether they were under the legal drinking age, or 21 and older. A young enlisted woman suggested:

It's just the way the Navy is made out to be, I guess. Sailors drink and so you drink. They gave you lots of opportunities to drink. There are a lot of functions and you know, everyone is getting together for beer after work, so okay, if everyone is going to go do it, I'm not going to be left out.

References to the ease with which a newly enlisted crewmember could find inexpensive alcohol and the planning of drinking groups were recurring themes throughout the qualitative interviews. In and around stateside bases, regardless of age, alcohol could be obtained without significant effort. For example, an enlisted female responded to the question, "What do people living on base do at night and weekends?" as follows:

I can tell you what I do. It's very easy to come out here and drink when you are underage. Out there on the strip. Anywhere! You can go in, off base. I was going, just bar hopping everywhere. I knew everybody knew me all over the place because, not that I was an alcoholic, but I would just keep going party, party, party, party. So a lot of people here are young and they are just coming out of high school, or it's the first time getting away from their parents and they all go crazy. All there is to do here basically, is there is a ton of bars.

It is worth noting that in our interviews with young enlisted personnel, we did encounter many mentions of official Navy discouragement of alcohol use, including providing non-alcoholic beverages at command functions. For example, referring to celebrations after playing on-base intramural sports, a young enlisted man said:

The Navy will give us the food or a certain amount of money, and we can use it to buy food and drinks, but it can't be alcoholic; my command doesn't allow for you to spend the Navy's money on anything alcohol related.

Despite this official emphasis on reducing social availability of alcohol, deeply embedded cultural traditions that support easy physical and social availability of alcohol in the work and social environments of our study population emerged as important risk factors for frequent heavy and binge drinking, especially given the largely underage drinking population of the sample.

### 2) On-base availability

#### Base clubs

Base clubs featuring live or deejayed music and alcohol have experienced a decline in popularity over the past twenty years in a number of the bases we visited. Reduced sales resulted in club closures and consolidations, which created situations where different parts of the same club catered to naval personnel of different ranks, reflecting the fraternization rules that discourage excessive informal interactions (including drinking) between members of different ranks. For example, one club had a separate bar explicitly limited to serving warrant officers, set aside from the larger space used by enlisted members.

Another reason for declining popularity of base clubs was elaborated by a civilian employee of an on-base club:

Bottom line is mainly your young GIs are men and there's just no women in there, so really nothing but their camaraderie and alcohol to keep them in there. So, until they get a lot of females in there it's going to their bar is not that busy. It's just the bottom line. I mean, man, they see each other all day, why do they want to see each other all night?

On some foreign bases, we also heard of young enlisted personnel being fearful of venturing into unfamiliar settings. This situational agoraphobic attitude fostered an insularity that encouraged forming on-base drinking groups, with young men and women participating in drinking parties in base clubs or their barracks in foreign bases. Such drinking networks reflect on-base social availability of alcohol use.

#### On-base exchanges – price and placement

Because base commissaries or Navy Exchanges sell products without taxes to base personnel, they constitute formidable competition to off-base retail outlets, offering lower prices on a variety of products, including alcohol. Although the campaign to deglamorize alcohol in the military (dubbed "The Right Spirit" in the U.S. Navy) requires that the exchanges limit alcohol promotions aimed specifically at servicemembers [37], we witnessed large pyramids of beer, and advertising just inside the entrances of several Navy Exchanges we visited. These beer displays were the first thing that a young person would see while shopping at the exchange. In response to the question, "If a young person who is over 21 wanted to buy alcohol here on the base, where would he get it?" a young enlisted male replied:

The Navy Exchange. Actually the commissary part of the Navy Exchange. They sell stuff in there and they got a pretty good sized selection of alcohol and beer. And it's definitely a lot cheaper than out in town because you don't have to pay a liquor tax or something that they put on it plus state tax.

In fact, the DoD Instruction for Military Exchanges states that "Prices of distilled spirits sold in the United States and the District of Columbia may be discounted no more than 10 percent less than the best local shelf price in Alcohol Beverage Control (ABC) States and 5 percent less than the best local shelf price in non-ABC States." [38]. However, with the tax-free status of exchange items, the actual price may be considerably lower than the maximum 10 percent deduction specified in the instruction.

#### Drinking in barracks – social availability

Clearly, the military has greater control over the underage drinking that occurs on base than in the communities off the base. The commanding officer has a certain degree of leeway in the amount of strictness he or she can impose in a disciplinary session (which is called Captain's Mast in the U.S. Navy, a term referenced by several of the following quotations). However, that control is not absolute, and the social availability of alcohol consumption as a form of relaxation during non-working hours is pervasive. Illustrating the contradiction between official alcohol control in the barracks and the actual practice of holding parties featuring alcohol for both underage and legal drinking age barracks residents, a young enlisted female said:

Living in the barracks, you constantly are reminded that you are in the military. They have a lot of rules: they have room inspections once a week; who wants to have your bedroom inspected to see if it's clean? "Oh you are a slob!" you know. You get into trouble for that kind of retarded stuff. You get ID'd to get into the barracks. It's just little things; it's like this is retarded, I am an adult. You can't drink in the barracks, you know. Like you're 21 and you are not allowed to have alcohol there. [And people don't?] Well I don't say that people don't...of course all rules are broken... Actually, they party a lot.

### 3) Off-base alcohol availability

Off-base, the settings for social availability of alcohol for naval personnel includes parties at homes, bowling alleys, clubs, and hotels. The following quotes offer a glimpse at the textured character of alcohol availability off-base.

[So when people do get together to drink, where do they go? You mentioned their rooms but do they ever go to clubs or off base to friends' homes?] Sometimes on weekends you go to parties at people's houses probably. Like we will go to a bowling alley. [On base?] If they are 21, you know. So a lot of people go there but the clubs around here, unless you are going to go to Chicago, they are icky. They are not very good clubs. They are very seedy. It's so expensive too. Then you have to find your way back on base. They're going to test you at the gate, you're going to get into trouble if you get caught; stuff like that.

As this young enlisted man noted, a key point of intervention that base authorities have over off-base activities is the security checkpoint at base entrances.

#### Off-base clubs

A noteworthy feature of many (but not all) bases are the bars and clubs located relatively close to the main gate of the base. We noted that it is not just the walking distance but also the visual impact of these establishments, with neon lights and open invitations to drink. In response to the question, "Where would young people be most likely to go drink? One of the places here on base, or off base?" a male officer replied:

That's geographical, but more likely here in Pensacola most people are going to be more apt to drink out in town. There's some great watering holes out in town. There's a big rise of people, you know, just going out, and just buying alcohol, and just finding a place to park and drink. That's been like that forever, but for a lot of folks, 'cause it's cheaper. I mean, you know, you figure the average E-4 (a petty officer, third class) and below just doesn't make a whole lot of money, so rather than pay 2 bucks a beer at a bar, you know, they spend 4 bucks and get a 6-pack. That all depends on the situation. In an area that's rural like this, that's perhaps pretty easy. Somewhere more like Virginia, it's probably easier to either go to the club, which is on base. Or they go to a club out in town.

Considering marital status as a key variable for involvement in drinking at off-base clubs, a submarine officer stated:

[How often, in work groups like yours, do young people go out drinking?] Well, guys in my division, it really depends on whether they're married or not, I think is the big factor. If you're a bachelor, and you're living in the barracks, you know, there's not much for you to do, but, everyone's going to go out and go to one of the clubs, I think, because you really have to get away from staring at the four walls of your hotel room, barracks room, whatever you want to call it.

In the post-9/11 security environment of military bases, opportunities for civilians to enter bases (and particularly submarine bases) in order to visit on-base clubs are limited. Therefore, naval personnel usually have to venture off-base in order to meet civilians socially.

Off-base social availability often reflects the desire of enlisted men and women to take a break from the barracks or ships where they reside, by spending some non-watch evenings in motels offering more comfortable beds and a greater degree of privacy. A young enlisted woman said that it was common to retreat to motel rooms when she had days off:

When you're living on board a ship, every time you get a paycheck, you try to go, just to get away from the ship so you can have somewhere to watch TV, sleep in a big bed... We get a paycheck on the first and the 15th. Right, I mean you have 2, 3, 4 people sharing a room.

The physical availability of alcohol off base includes low prices and drink specials, including some exclusively for the military. A young enlisted man identified some nights that are more popular for going out drinking:

It's probably more Thursdays, Fridays and Saturdays. In [a bar featuring country-western music], they have military night where military gets in free and drinks $2.50 pitchers all night. So there's a lot of military that goes in there. It's only 3 bucks to get in. That's pretty cheap, actually. [Drinks at] most clubs are between 5 and 8 bucks. That's why I don't go out much unless it's on Thursday night because it's free for the military to get in and $2.50 pitchers all night long.

An officer identified specific bars in San Diego featuring drink specials and said that he had noticed that groups of naval personnel would traverse the circuit of bars with such specials:

In Pacific Beach, depended like night to night where you would go, where the specials were, and after a while, I didn't know them personally, but, I would start to recognize the same people depending on what night of the week it was, because, you know, happy hour this night at this bar or whatever You know, no cover, $1 beer 'til 10:00 pm, get people in the bar.

Clearly, the owners of the bars surrounding naval bases are aware of the limited means of junior enlisted personnel, and offer cover and drink specials in order to encourage them to frequent their establishments.

### 4) Underage issues

Although underage purchase laws are adhered to by most bars located near U.S. bases, underage enlisted personnel reported that it was not difficult to obtain alcohol in bars, in the Navy Exchange, barracks, or hotel rooms near the base. If underage, friends could provide the alcohol. For example an enlisted male, 22 years old, discussing when he was underage, said:

[Where would you get the alcohol?] From on base, the store. [Even though you weren't 21?] No, I'm sorry, we wouldn't be able to buy it, whoever in our barracks is old enough, they could go out and buy it, and come back to the barracks and we'd drink it.

On-base stores and exchanges typically do ask young purchasers of alcohol for identification, but respondents stated that, as in other contexts, clerks may not continue the practice with familiar people. In response to a question regarding buying alcohol on base, a young enlisted man said:

You can buy it at the exchange and there's a comm. (commissary) and mini-mart down here by Subway on the Sea Wall. You can buy alcohol. [What about under 21? Do they card here?] They do, but they know the regular people that buy, they know, if they've carded you a couple of times, they know who you are, and they get to know who you are and they just kind of say "Okay."

Although in the past, some commanding officers in bases close to foreign borders relaxed the age 21 drinking limit to 18 for young enlisted personnel, the guidelines spelled out by section 6.3 of the Department of Defense Instruction 1330.21 [37] tightened age-based restrictions throughout the United States bases. As a result, most bases have more strictly upheld underage drinking laws. An officer in his late 20s who supervises young enlisted men and women stated:

It used to be a couple years ago, when I was on midshipman cruise out in San Diego, that, if you were on base, and you were 18 years old, you could drink, as long as you didn't go off base drinking, that was fine. They've gotten rid of that rule, that law pretty well. I don't think it happens anymore at all, but, yeah, there's like, Club Metro (on base), all those bars that are available.

A male petty officer said that the way in which someone under 21 would obtain alcohol is as follows:

Ask someone who was over 21 to buy it for them. People are going to get into real serious trouble for it but it happens. I am not going to say it doesn't happen because I know it does.

Sympathetic servers believing that underage soldiers and sailors should be allowed to drink, as well as those accepting false identification, will sell alcohol to minors serving in the military.

When asked how underage enlisted personnel get alcohol, a 19 year old male responded:

A lot of people will take that military ID, "oh, you are old enough to defend the country, go ahead". A lot of people have hook up; they know people this and that, whatever. As far as bars and stuff, downtown San Diego, I think they are pretty strict, but I think a lot of people probably have fake ID's.

Moreover, on base, friends or co-workers who are 21 may buy alcohol for underage colleagues in barracks. A young enlisted man replied to a question about where he would obtain alcohol in the following way:

From on base, we wouldn't be able to buy it; whoever in our barracks is old enough, they could go out and buy it, and come back to the barracks and we'd drink it...usually beer.

A young enlisted woman said that she would not drink on base:

Yeah, they're really strict about here, and even being under 21, I mean, I'm under 21 right now, and I mean I drink but not on base, you know same thing I'll drink at home, I'll have a few beers, or I'll drink a beer or something, you know? But I don't think Anybody would be really stupid to drink on base knowing you're underage, get caught, nuh-uh. Captain's Mast right there.

#### Tijuana

A special case of underage drinking we heard frequently in San Diego naval bases was the border crossing for Tijuana. When we asked a young enlisted woman if she thought her shipmates drank the same amount they did before they joined, she replied:

No, I think it's probably more. I think it's because of 1) boredom 2) they don't want to stay on base during their liberty, so, if you're young, you could either go to Tijuana, that's 18 and above drinking age. If you're under 21, then you could always go to Tijuana and drink and then come back, or, if you are 21, it's just pretty much to get off base.

What makes Tijuana particularly appealing to San Diego-based naval personnel under 21 years of age are the lower age limit and price offered by the bars across the border. In response to a question about going to bars after work, a San Diego-based young enlisted man said:

Most people go home, but a lot of guys, here, because it's so close to Tijuana, usually they'll go home for a little while, and then do the drinking thing. [Because it's cheaper or because they're under 21?] Well, I think that the guys that are under 21 go down there because they are under 21, but the guys that are over 21 go there, usually it's because of the price. I hear stories all the time that you go down there and to certain clubs, for $7 you can drink all night.

In response to the problems associated with underage naval personnel going to Tijuana to get intoxicated, some naval authorities in San Diego instituted a policy whereby possession of a permission slip or chit from one's supervisor was required to cross the border. A young enlisted woman said that her San Diego-based shipmates who were under 21 were tempted to go to Tijuana:

Go down there at 18; all you have to do is be 18. But, they get in trouble when they are coming back. Our ship has a policy you have to have a special request slip even to go down there. It is a foreign country. If you don't have that request slip and you get caught, you go to Captain's (Mast).

As the foregoing quotes suggest, whether or not underage military personnel are close to a border town with lower drinking ages, the social availability of alcohol among their peers is generally high.

### Survey findings regarding alcohol availability

Quantitative findings regarding alcohol availability from a survey of 713 enlisted men and women provide additional evidence for abundant availability of alcohol both on and off base for naval personnel aged 21 and over as well as for their underage counterparts. Table 1 compares the percentage of underage respondents with those of drinking age who responded that obtaining alcohol was either "easy" or "very easy" in a variety of situations, including on and off base after work, and from friends in and outside of the Navy. In each case, the majority of responses suggested the perception of considerable ease in obtaining alcohol.

**Table 1:** Survey findings on alcohol availability for underage and legal age enlisted naval personnel. Numbers may not add up to 100% due to rounding.

	Underage	Legal Age	χ^2^	p-value
**Subjective Physical Availability**				
Obtaining Alcohol on Base after Work:				
Easy/very easy	63%	81%	26.91 2df	<0.001
Difficult/very difficult	26%	11%		
Don't know	11%	8%		
Obtaining Alcohol off Base after Work:				
Easy/very easy	80%	90%	12.05 2df	0.002
Difficult/very difficult	12%	6%		
Don't know	8%	4%		
				
**Subjective Social Availability**				
Obtaining Alcohol from Friends in the Navy:				
Easy/very easy	88%	85%	1.79 2df	0.41
Difficult/very difficult	7%	9%		
Don't know	5%	6%		
Obtaining Alcohol from Friends outside the Navy:				
Easy/very easy	84%	81%	1.67 2df	0.43
Difficult/very difficult	8%	10%		
Don't know	8%	10%		

In terms of subjective physical availability, there were significant differences (χ^2 ^= 26.91, 2 df, *p *< .001) between underage respondents and those of legal drinking age regarding ease of obtaining alcohol on base after work. For example, 63% of underage respondents said that it was easy or very easy to obtain alcohol on base after work, compared to 81% of legal drinking age respondents. About 26% of underage drinkers said it was difficult or very difficult, compared to 11% of legal age drinkers. Similarly, there were significant differences (χ^2 ^= 12.05, 2 df, *p *= .002) between underage drinkers and legal drink age respondents concerning ease of obtaining alcohol off base after work. A smaller proportion of underage respondents than those of legal drinking age (approximately 80% vs. 90%) reported that it was easy or very easy to obtain alcohol off base after work. About twice as many underage respondents than legal drinking age respondents (12% vs. 6%) stated it was difficult or very difficult. Despite the fact that respondents 21 and over find it easier to purchase alcohol, a substantial percentage of underage drinkers find it easy to obtain alcohol as well.

Subjective social availability was indexed by the questions regarding obtaining alcohol from friends. There were no significant differences between underage respondents and those of legal drinking age concerning ease of obtaining alcohol from friends in the Navy (χ^2 ^= 1.79, 2 df, *p *= .41) or from friends outside of the Navy (χ^2 ^= 1.67, 2 df, *p *= .43). In either case, only a small minority (7.7% and 8.5%, respectively) thought that it would be difficult or very difficult to obtain alcohol from friends. Thus most respondents shared the view that subjective social availability was high.

## Discussion

There is qualitative and quantitative evidence for extensive physical and social availability of alcohol in and around bases for young adults in the military. In terms of the physical availability of alcohol, it is inexpensive and easy to obtain, both in base exchanges and commissaries, and in drink promotions at nearby off-base clubs. More generally, despite the movement to deglamorize alcohol within the military, pervasive and longstanding cultural tradition of drinking is the default activity for enlisted personnel to pursue when there is no other perceived attractive recreational alternative (despite the extensive offerings of the Navy's Morale, Welfare and Recreation program, such as auto repair workshops and sports leagues that are financially supported by sales at the base exchanges). The potential for socializing with women in off-base establishments constitutes another draw for many enlisted men to frequent off-base bars and clubs.

Other drinking settings mentioned in our interviews, particularly by underage enlisted respondents, included parks, hotel rooms, and the homes of friends; these environments fall squarely within the realm of social availability. Tijuana was mentioned several times as the nearby attraction for underage drinkers serving in San Diego because it is legal to drink at age 18 in Mexico [39], and for others of age (21+) because of the low prices. Falsified identification cards for underage drinkers was mentioned on several occasions; this is a more widespread issue [40] which suggests that investments in technology to detect false IDs may be warranted in order to reduce their effectiveness.

This study is distinctive for several reasons. First, we have given focused attention to subjective and empirical dimensions of alcohol availability in military base settings. Drawing from definitions of alcohol availability as applied to the general population, and from relevant findings from studies of occupational drinking, we have presented definitions for physical and social availability of alcohol as applied to the military base population.

Second, results of this study are applicable for prevention, and we suggest that prevention programs give more attention to reducing environmental support for heavy drinking on and around military bases. Once this information is disseminated and understood among military leadership and the population of young adults, a unified effort can be made to make changes in the base environment and policies for off-base outlets, thereby reducing risk factors that lead to heavier and problem drinking among young military personnel.

## Conclusion

As a number of quotes from the respondents in this study have illustrated, the availability of alcohol, both social and physical, is only slightly diminished for underage Navy personnel relative to their counterparts of legal drinking age. Although there are barriers to directly obtaining alcohol on base, having over-21 friends purchase alcohol for on-base parties as well as wider off-base opportunities for direct purchases from sympathetic servers or using false IDs permits extensive drinking by underage enlisted men and women.

The theoretical contribution of this paper is to extend Ames and Grube's [24] definition of work-related alcohol availability to military and other on-call populations. This paper explicates how work-related alcohol availability in the military includes after-work drinking in and around bases; such alcohol consumption is shaped by military culture and affects troop readiness to respond at any time.

Policy implications of this study include raising alcohol prices in base stores to reflect the actual prices, including taxes, of off-base outlets and enforcing existing policies regarding the provision of alcohol to minors in and around bases – particularly those concerning furnishing alcohol to underage comrades. The imposition of limits on servicemembers' travel across borders to areas with virtually unrestricted access to alcohol represents a promising solution to that channel of underage availability [41]. Within their bases, at least, officers and senior enlisted have the power to bolster existing Navy efforts to offer engaging alcohol-free events to enlisted personnel, leveraging their experience with providing non-alcoholic beverages at command functions. We acknowledge that altering long-standing practices and economic systems of alcohol supply in the communities both on and off base [42] represent a substantial challenge for the commanding officers who are implementing the concept of alcohol deglamorization promoted by the Navy's leadership [4].

Because the surrounding communities play an important role in providing alcohol to young adults, but also experience the consequences of their excessive consumption (e.g., fights, property damage, and instances of driving under the influence), partnerships between base management and neighboring community coalitions [42] can help to apply meaningful changes in alcohol availability around bases. Reduction of underage drinking is a common area on which the bases and the surrounding communities may jointly focus. For example, improving detection of false IDs [43] and engaging in server training [44] and enforcement efforts to address the phenomenon of sympathetic serving to underage military personnel are some areas where base leadership and communities can work together. Such cooperation is not merely theoretical: Recently, the U.S. Air Force has partnered with communities surrounding bases [45], implementing a warning to off-base bars that if they are caught serving alcohol to minors, they will be declared off-limits to all base personnel.

In these conclusions, we do not differ markedly from those reached by Kuo et al. [13] in their study of alcohol availability on college campuses. As another population of young adults with rich traditions surrounding the use of alcohol to celebrate and relax, college students provide a useful parallel to young adults in the U.S. Navy. A critical difference between these populations, however, remains the work-related character of alcohol availability for these young men and women serving in the military; the fact that they are always subject to immediate recall for duty places the consequences of heavy and problem drinking related to high work-related availability of alcohol in a different category from students. The public health implications for availability-facilitated long-term drinking patterns and the related health and social consequences are nevertheless equally important for students and enlisted personnel alike.

## Competing interests

The author(s) declare that they have no competing interests.

## Authors' contributions

RSM carried out the qualitative analysis presented here, helped to write the study proposal, conducted many of the interviews and drafted the manuscript. GMA was the Principal Investigator of the overall study, also conducted many of the interviews, and helped to analyze the interview data and draft the manuscript. CBC led the development of the survey and conducted and wrote up the survey analyses reported on here. All three authors read and approved the final manuscript.
